# The WHO BMI System Misclassifies Weight Status in Adults from the General Population in North Italy: A DXA-Based Assessment Study (18–98 Years)

**DOI:** 10.3390/nu17132162

**Published:** 2025-06-29

**Authors:** Chiara Milanese, Leila Itani, Valentina Cavedon, Marwan El Ghoch

**Affiliations:** 1 Department of Neurosciences, Biomedicine and Movement Sciences, University of Verona, 37129 Verona, Italy; chiara.milanese@univr.it (C.M.); valentina.cavedon@univr.it (V.C.); 2 Department of Nutrition and Dietetics, Faculty of Health Sciences, Beirut Arab University, Riad El Solh, Beirut P.O. Box 11-5020, Lebanon; l.itani@bau.edu.lb; 3 Center for the Study of Metabolism, Body Composition and Lifestyle, Department of Biomedical, Metabolic and Neural Sciences, University of Modena and Reggio Emilia, 41125 Modena, Italy

**Keywords:** adiposity, body fat, body mass index, body composition, public health, weight status

## Abstract

**Background/Objectives**: The body mass index (BMI) is considered a key method for the classification of individuals’ weight status, according to cut-off points proposed by the World Health Organization (WHO); however, the use of this classification is still the subject of debate and criticism. We aimed to evaluate the accuracy of the WHO BMI classification in reflecting true adiposity in the Italian general population. **Methods**: This cross-sectional study included 1351 adults of mixed gender aged between 18 and 98 years, comprising 19 (1.4%) underweight individuals, 787 (58.3%) normal weight, 354 (26.2%) overweight, and 191 (14.1%) with obesity according to the WHO BMI. After that they were re-categorized according to adiposity based on body fat percentage (BF%) measured by dual-energy X-ray absorptiometry (DXA). The agreement between the two classification systems was tested using the kappa statistic (κ), with the system based on BF% being considered the gold standard. **Results**: According to the BMI classification, 78.1% of the individuals who were in the normal weight range were correctly classified. However, 53.4% of the overweight group and 68.4% of the underweight group were misclassified according to the BMI, as the majority of those misclassified fell within the normal weight range according to their BF%. Finally, regarding the obesity group, 34% who were classified as having obesity according to the BMI were misclassified, since they were revealed to be only affected by overweight according to adiposity status. **Conclusions**: Despite the fact that the BMI seems to be reliable in determining body weight status in the normal weight range, over a third of the general population was misclassified, as the current BMI classification appears to inflate the prevalence of underweight, overweight, and obesity among the general population. Accordingly, this may warrant consideration of revising the National Guidelines in Italy related to weight status classification. Healthcare practitioners should be advised not to rely solely on the BMI, and should integrate its use with adiposity measures (i.e., BF%) or alternative surrogate indicators (i.e., waist-based) in routine evaluations, especially in those with a BMI below or above 18.5 kg/m^2^ or 25 kg/m^2^.

## 1. Introduction

The body mass index (BMI) is the ratio between body weight, expressed in kilograms, and squared height in meters [1]. It remains the most used indicator of health, and risks of morbidity and mortality, at both clinical and epidemiological levels [2,3]. Scientific bodies such as the World Health Organization (WHO) [4], the Centers for Disease Control and Prevention (CDC) [5], the National Institute for Health and Care Excellence (NICE) [6], and many others, rely on BMI cut-off points (i.e., ≥18.5 kg/m^2^, 25 kg/m^2^ and 30 kg/m^2^) to classify weight status to indicate underweight, normal weight, overweight, and obesity, respectively, for adults in almost all age, gender, and ethnicity groups.

The BMI is considered a surrogate measure to determine thinness or fatness [7], usually associated with malnutrition and several chronic diseases [8]. However, to this aim, BMI categories are not precise when compared to adiposity classification based on direct body fat measurements, which is considered the gold standard [9], or other alternative surrogate measures, especially waist-based indicators (i.e., waist-to-height ratio), which have proven to be more associated with several chronic diseases than the BMI [10]. This last fact relates to the classification categories of the BMI relying on broad ranges that are not widely accepted due to several limitations [11,12]. Foremost, the BMI does not account for body composition compartments (i.e., muscle and fat) in terms of quantity or distribution. Nor does it account for changes with aging during the human lifespan [13], characterized by an increase in total and visceral fat, as well as a decrease in lean mass (i.e., sarcopenia), which may occur due to physiological fluctuations in body weight but with no significant changes in the BMI [13,14]. These changes in body composition compartments are really the determinates of wellbeing and predictors for the risk of having diseases, as well as clinical outcomes [15,16]. Moreover, in several specific and general population groups, inconsistent associations were found between BMI classification and levels of adiposity [17]. It has been demonstrated that a BMI falling within normal ranges may mask an underweight condition [18], and also overweight or obesity (i.e., normal weight obesity), and vice versa [19]. In fact, recent studies suggest the determination of weight status by BF% instead of BMI may be more instructive, especially for those with overweight and obesity [20]. Similarly, the WHO BMI classification system of weight status may also have a societal impact, e.g., the cost of life insurance premiums, as many companies still rely on BMI as a primary health metric. Indeed, people with a BMI in the overweight (25–29.9 kg/m^2^) or obesity (≥30 kg/m^2^) ranges may face higher insurance premiums because they are considered to be at greater risk for serious health problems [21]. In fact, individuals can see themselves rendered uninsurable if they fall beyond certain BMI cut-off points (i.e., <18.5 kg/m^2^ or ≥40 kg/m^2^).

Based on all these considerations, the current study aims to investigate to what extent the WHO BMI cut-off points for underweight, normal weight, overweight, and obesity classifications (i.e., 18.5, 25, and 30 kg/m^2^, respectively) are accurate for use as an indicator of understated, normal, overstated, and excess adiposity in a general population composed of adults aged between 18 and 98 years in North Italy. Within this scope, the hypothesis is that the WHO BMI classification in our population will misclassify the weight status when compared to that determined by adiposity level.

## 2. Materials and Methods

### 2.1. Participants and Design of the Study

This study is of a cross-sectional observational design conducted in adherence to Strengthening The Reporting of Observational Studies in Epidemiology (STROBE) [22]. The participants were randomly recruited to take part in the study from university listservs of students, and current and retired employees, by email, as well as online advertisements addressed to the general population. The participation rate was calculated as the number of those who agreed to participate divided by those invited; in our case the participation rate exceeded 60%, which is considered reasonably acceptable for epidemiological studies [23]. Those who were referred to the Department of Neurosciences, Biomedicine and Movement Sciences, University of Verona, Verona, Italy, were enrolled during the period between January 2013 and December 2024. The inclusion criteria involved (i) having an age ≥18 years; (ii) being of a white Caucasian ethnicity; and (iii) having completed a body composition measurement using DXA. Participants were excluded if they were (i) children or adolescents aged less than 18 years; (ii) of a different ethnicity (i.e., Black or Non-Hispanic Asian); (iii) pregnant (among females); (iv) taking medication that affects body weight or composition; or (v) affected by medical comorbidities associated with weight loss or gain such as cancers and severe psychiatric disorders. A total of 1351 individuals of mixed gender and four age groups—young (18–39 years), middle-aged (40–59 years), older (60–79 years), and oldest (80+ years)—with different body weight statuses according to the WHO BMI classification of underweight (*n* = 19), normal weight (*n* = 787), overweight (*n* = 354), or obesity (*n* = 191) were included.

This research adhered to the Declaration of Helsinki and was approved by the Institutional Review Board (IRB) at the University of Verona (Prot. No. 2012-290). All participants’ personal data were treated according to European/Italian privacy laws, and informed written consent was obtained.

### 2.2. Body Weight and Height

Body weight was assessed to the nearest 0.1 kg with an electronic scale (Tanita electronic scale BWB-800 MA) and height was measured with a Harpenden stadiometer (Holtain Ltd., Crymych, Pembs, UK) to the nearest 0.01 m [24]. Both measurements were taken with the subject wearing no shoes and minimum clothing. The BMI was then calculated according to the standard formula of body weight measured in kilograms divided by the square of the height in meters.

### 2.3. Body Composition

Total and segmental body composition were determined by means of a DXA scanner (QDR Explorer W; Hologic, MA, USA; fan-beam technology, software for Windows XP version 12.6.1) according to the manufacturer’s instructions [25]. To avoid possible baseline drift, the scanner was checked daily against a standard anthropomorphic spine phantom supplied by the manufacturer and all scans were performed by the same operator in order to ensure consistency. During the test, the participants were given complete and standardized instructions on the testing procedure, as described elsewhere, and they wore lightweight clothing with no metal or reflective material, and removed all metal accessories [26]. Due to the well-documented changes in body composition compartments in all weight status categories, namely an increase in BF and a decrease in muscle mass across age and gender groups in all body weight categories [13,14], the sample was categorized according to the widely validated adiposity classification system based on age- and gender-specific underweight, normal weight, overweight, and obesity BF% cut-off points as follows [27]:

For males:

- 18–39 years BF% < 8% (underweight); ≥8% (normal weight); ≥21% (overweight; ≥26% (obesity);
- 40–59 years BF% < 11% (underweight); ≥11% (normal weight); ≥23% (overweight; ≥29% (obesity);
- 60–98 years BF% < 13% (underweight); ≥13% (normal weight); ≥25% (overweight; ≥31% (obesity).

For females:

- 18–39 years BF% < 21% (underweight); ≥21% (normal weight); ≥33% (overweight; ≥39% (obesity);
- 40–59 years BF% < 23% (underweight); ≥23% (normal weight); ≥35% (overweight; ≥41% (obesity);
- 60–98 years BF% < 26% (underweight); ≥26% (normal weight); ≥36% (overweight; ≥41% (obesity).

### 2.4. Statistical Analysis

Descriptive statistics are presented as means and standard deviations for continuous variables and frequencies and proportions for categorical variables. Welch’s test was used to compare means for continuous variables to correct for unequal variances where necessary. Given the ordinal nature of the BMI and BF classification systems, a weighted kappa was used to measure agreement [28,29]. Kappa values ≤ 0 indicated no agreement, 0.0–0.2 none or slight, 0.2–0.4 fair, 0.4–0.6 moderate, 0.6–0.8 substantial, and 0.8–1.00 an almost perfect agreement [30,31]. Misclassified participants were coded within each BMI range/category by assigning 0 for those classified in the right category based on BF%, and 1 for those misclassified. The frequency of misclassification was then determined across sex, age, and BMI categories. The chi-squared test for independence was used for categorical variables to assess the distribution across BMI and age categories. The distribution of misclassified individuals is graphically presented using scatter plots. All analysis was conducted using SPSS ver. 27 [32] and NCSS (ver. 23.0.2) [33].

## 3. Results

The overall study sample included 1351 adult participants, with 60% females (*n* = 810) and 40% males (*n* = 541). The overall mean age was 45.5 ± 21.4 years, with males being older (49.7 ± 21.7 vs. 42.7 ± 20.7 years). The overall mean BMI was 25.0 ± 4.6 kg/m^2^, with males being 1.3 BMI units heavier than females (25.8 ± 3.9 vs. 24.5 ± 5.0 kg/m^2^). Males had higher appendicular lean mass (ALM) (25.1 ± 3.5 vs. 17.4 ± 2.9 kg), but significantly lower BF (17.8 ± 7.4 vs. 20.5 ± 8.8 kg) and BF% (22.5 ± 6.7 vs. 31.2 ±8.1%) (Table 1).

The prevalence of different body weight status in the overall sample based on the BMI and BF% classification systems is presented in Table 2. According to the WHO BMI classification, the prevalence of underweight in the overall sample was minimal (1.4%), with 58.3% being of normal weight, 26.2% being overweight, and 14.1% being obese. Based on the BF% classification (Gallagher criteria), the prevalence of under fat (i.e., underweight) in the overall sample was 6.2%, with 57.2% having normal fat (i.e., normal weight), 23.4% over fat (i.e., overweight), and 13.2% having excess fat (i.e., obesity).

The two classification systems resulted in similar total rates for each weight status category (Table 2); however, considering the agreement between the two classification systems within each category, a significant disagreement is clearly observed (Table 3). Since adiposity is best defined by means of BF%, the comparison was computed using BF categories as a reference for agreement analysis. In this respect, the BMI classification captured only 31.6% of those who were underweight, 78.1% having normal weight, 46.6% of those who were overweight, and 66.0% of those with obesity based on BF%. Weighted kappa (*k* = 0.126) revealed a significant weak agreement between the two classification systems (i.e., BMI vs. BF%).

**Figure 1:**
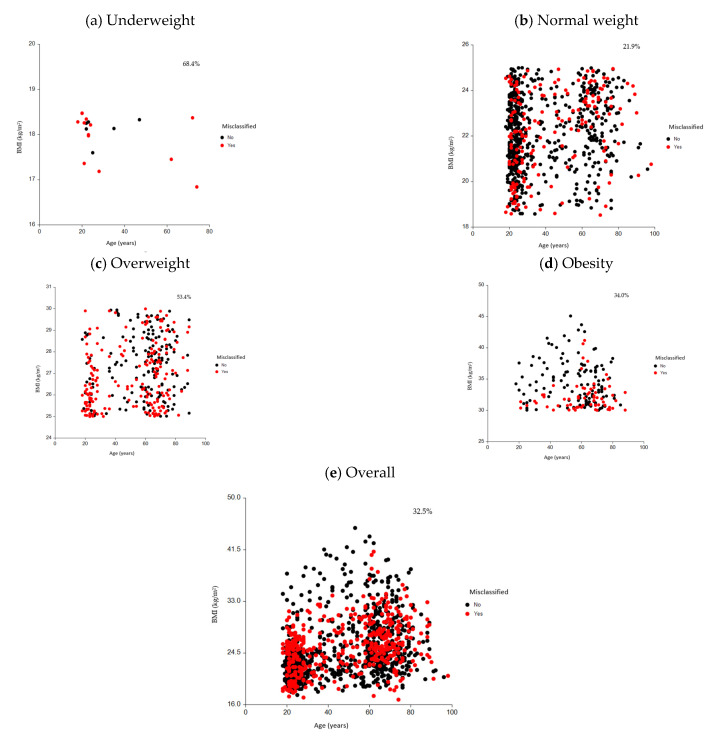
Distribution of misclassification of weight status based on BMI classification system in (**a**) underweight; (**b**) normal weight; (**c**) overweight; (**d**) obesity and (**e**) overall groups.

To assess the magnitude of misclassification, the distribution of misclassification by BMI category, sex, and age group is presented in Table 4 and Figure 1a–e. In the overall population, the rate of misclassification observed was 32.5%, with similar rates among males (32.7%) and females (32.3%). Looking at the distribution of misclassification by age group, in the overall sample, around two-fifths (42.1%) of young adults (18–39 years), 13.9% of middle-aged adults (40–59 years), around two-fifths (39.0%) of elderly adults (60–79 years), and 5.0% of oldest adults (80+ years) were misclassified. For females, almost half (48.1%) of young adults (18–39 years), 15.6% of middle-aged adults (40–59 years), less than one-third (30.2%) of elderly adults (60–79 years), and 6.1% of oldest adults (80+ years) were misclassified. For males, one-third (33.3%) of young adults (18–39 years), 11.3% of middle-aged adults (40–59 years), more than half (52.0%) of elderly adults (60–79 years), and 3.4% of oldest adults (80+ years) were misclassified.

The prevalence of misclassification within BMI categories ranged from 21.9% in the normal BMI group to 68.4% in the underweight BMI group. Looking at the distribution of misclassification by age group for those within the underweight BMI range, almost two-thirds (66.7%) of young adults (18–39 years) were misclassified, and 100% of older adults. For those within the BMI normal weight range, around a fifth (19.3%) of young adults (18–49 years), a quarter (24.7%) of middle-aged adults (40–59 years) and older adults (60–79 years), and almost half (47.4%) of oldest adults (80+ years) were misclassified. Among those within the BMI overweight range, more than two-thirds (69.6%) of young adults (18–39 years), two-fifths (41.1%) of middle-aged adults (40–59 years), and close to a half (48.6% and 47.1%) of older (60–79 years) and oldest (80+ years) adults, respectively, were misclassified. Among those within the BMI obesity category, more than a quarter (27.3%) of young adults (18–39 years) and middle-aged adults (40–59 years) (28.8%), nearly two-fifths (37.1%) of older adults (60–79 years), and more than a half (55.6%) of oldest adults (80+ years) were misclassified in this BMI category.

Among the females, the highest rate of misclassification observed in descending order was among the BMI underweight range (60.0%), followed by overweight (54.8%) and obesity (34.2%), with the least misclassification among those within the normal weight BMI (24.4%) category. Looking at the distribution of misclassification by age group, for females within the BMI underweight range, almost two-thirds (64.3%) were misclassified among young adults (18–49 years), with no data for the other age groups. For those within the normal weight BMI range, around a quarter (23.4%) of young adults (18–39 years), more than a quarter (28.4%) of middle-aged adults (40–59 years), around a fifth (22.4%) of older adults (60–79 years), and around two-fifths (42.9%) of oldest female adults (80+ years) were misclassified. Among those within the BMI overweight range, more than two-thirds (67.3%) of young adults (18–39 years), almost a third (28.6%) of middle-aged adults (40–59 years), more than a half (57.6%) of older adults (60–79 years), and more than two-thirds (71.4%) of oldest female adults (80+ years) were misclassified. Among those within the BMI obesity category, around a fifth (18.2%) of young adults (18–39 years), more than a quarter (26.3%) of middle-aged adults (40–59 years), around two-fifths (41.7%) of older adults (60–79 years), and more than four-fifths of (83.3%) oldest female adults (80+ years) were misclassified.

Among the males, the highest rate of misclassification observed in descending order was among the BMI underweight range (100%), followed by overweight (52.3%) and obesity (33.8%), with least misclassification among those within the normal weight (16.7%) BMI category. Looking at the distribution of misclassification by age group, for males within the BMI underweight range, all (100%) were misclassified among young adults (18–39 years) and older adults (60–98 years), with no data for other age groups. For those within the BMI normal weight range, around a tenth (9.9% and 10.5%) of young adults (18–39 years) and middle-aged adults (40–59 years), respectively, more than a quarter (27.3%) of older adults (60–79 years), and around two-thirds (60.0%) of oldest male adults (80+ years) were misclassified. Among those within the BMI overweight range, more than two-thirds (71.7%) of young adults (18–39 years), almost two-thirds (61.9%) of middle-aged adults (40–59 years), more than two-fifths (43.4%) of older adults (60–79 years), and around a third (30.0%) of oldest male adults (80+ years) were misclassified. Among those within the BMI obesity range, around a half (45.5%) of young adults (18–39 years) and more than two-thirds (35.7% and 32.7%) of middle-aged (40–59 years) and older (60–79 years) adults, respectively, and none among oldest male adults (80+ years) were misclassified.

## 4. Discussion

The current study aimed to test the reliability of the WHO BMI cut-off points in classifying the weight status of individuals in the general population compared to DXA-derived adiposity levels. Three main findings were revealed as follows.

### 4.1. Findings and Concordance with Previous Studies

Firstly, on a general scale, our main finding highlights the fact that a large proportion of individuals, exceeding one-third of the Italian general population, have a misclassified weight status when relying on the traditional WHO BMI classification when compared to the classification based on adiposity levels according to BF%. Our finding is in line with previous studies regarding the inaccuracy of use of BMI in both clinical samples [9,20,34,35,36] or certain specific populations (i.e., athletes, soldiers, etc.) [37,38].

**Figure 2:**
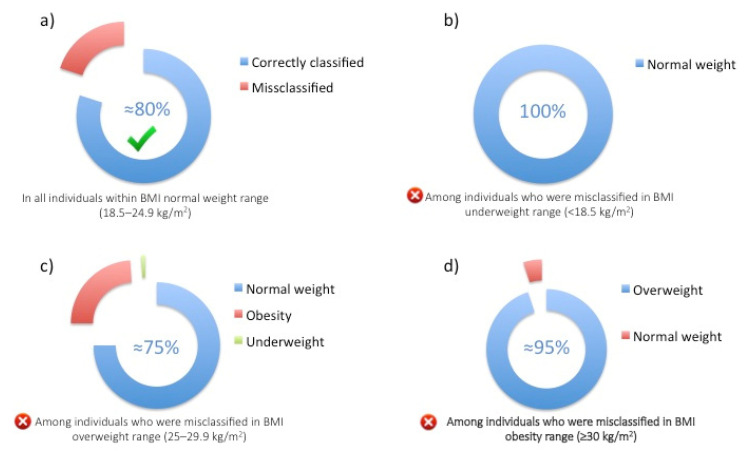
Misclassification constituents according to BF% within BMI ranges. (**a**) The proportions of individuals who are correctly classified (blue) or misclassified (red) in the BMI normal weight range; (**b**) The proportion of individuals with normal weight among those misclassified in BMI underweight range; (**c**) The proportions of individuals with underweight, normal weight or obesity among those misclassified in BMI overweight range; (**d**) The proportions of individuals with normal weight or overweight among those misclassified in BMI obesity range.

Secondly, analyzing our results in depth (especially when taking BMI categories separately), with regard to the normal weight range, the BMI classification can still be considered a reliable tool to identify to a high extent (≈80%) individuals that fall correctly within this weight status (Figure 2a). Among those classified as normal weight according to the BMI classification, the majority were also confirmed as correctly classified based on BF classification for both genders and in all age groups, except for those who are in the oldest age group. This result appears to contrast with consistent findings in the literature that claim that the BMI normal weight range may mask individuals with overweight or obesity. In our sample, however, only 10% were overweight according to BF%, and less than 1% with obesity, contrasting with the concept of normal weight obesity in this population [39].

Thirdly, 70% of those who fell in the underweight BMI range were wrongly classified, as all these individuals were effectively normal weight according to their adiposity level based on the BF% classification (Figure 2b). Moreover, 50% those of who were classified in the overweight BMI range were not, as the majority (75%) of the misclassified were still normal weight (Figure 2c). Finally, 35% of the individuals who were classified as having obesity according to BMI were only affected by overweight according to their BF% (Figure 2d). Therefore, the WHO BMI classification seems to inflate rates of abnormal weight status as being under- or overweight where in reality they are normal weight, or even categorizing obesity when individuals are in fact overweight. This finding, in particular, should be interpreted with extreme caution since it goes against the current available evidence, which reports the high prevalence of normal weight individuals among underweight and overweight BMI classifications (i.e., BMI < 18.5 kg/m^2^ and ≥25 kg/m^2^, respectively), as well as overweight individuals who are misclassified as being affected by obesity by having BMI ≥ 30 kg/m^2^. However, to date, these findings, especially the one regarding the overestimation of the prevalence of overweight or obesity rates, have already been reported by previous investigations conducted in other general populations (i.e., US, Canada, and Middle East) [40,41,42]. However, this finding still requires further replication before drawing any firm conclusion.

### 4.2. Strengths and Limitations

Our study has several strengths. To the best of our knowledge, it is one of the few evaluations, if not the first, to test the validity of the WHO BMI cut-off points (i.e., 18.5, 25, and 30 kg/m^2^) to determine weight status in a large group of individuals composed of young, middle-aged, older, and oldest effectively healthy adults in a “real-world” general population setting in Italy. Second, body composition was measured using DXA, which is known to exhibit a high level of precision, especially in the assessment of BF in different weight status groups in lean people or those affected by overweight or obesity [43,44]. Our investigation, however, also had some limitations. Most importantly, the data were obtained from one region in Italy, so external validation in others is necessary [45]. Secondly, some subgroups included in our study, such as those under 18.5 kg/m^2^, as well as those in the oldest age group, were small samples; therefore, findings derived from those should be interpreted with caution [46]. Thirdly, the limitations of certain statistical analysis tests that were used (i.e., Cohen’s kappa test) may have some implications regarding sensitivity to the prevalence of agreement in the data. In particular, when the categories being rated are imbalanced or when there is a high prevalence of one category, this test tends to be biased and may not accurately reflect the true agreement between raters [47]; however, this was not the case in our study, since no imbalanced rates were reported among the different categories. Fourthly, several lifestyle factors that may negatively or positively influence body composition compartments regardless of BMI levels, such as physical activity levels [48], diet and food intake [49], and sleeping [50] and smoking [51] habits, as well as socio-demographic and biochemical variables, were lacking in our study; therefore, we were not able to assess their impact on weight status. Finally, our study was of a cross-sectional design; therefore, it was unable to detect BMI and body composition trends or changes, which usually requires longitudinal assessment [52].

### 4.3. Clinical Implications and New Directions for Future Research

If confirmed, our findings have several clinical implications. Firstly, the National Health Service, policy-makers, the health insurance system, and other societal bodies (i.e., companies, schools, etc.) in Italy which still rely on BMI as a health indicator are invited to take our results as at least preliminary evidence for considering that the WHO BMI classification system is only reliable for identifying normal weight status in the general population. Critically, the same cannot be said for underweight, overweight, or obesity. Secondly, awareness should be raised among all healthcare professionals, especially in primary healthcare settings (i.e., general practitioners) during weight status assessment by means of BMI, of its limitations. In particular, relying solely on it may inflate the prevalence of underweight, overweight, and obesity, and thus targeting these individuals as having unhealthy weight status and assessing them as requiring weight management and treatment may be inappropriate. This would certainly translate into additional medical expenses that can overwhelm national healthcare systems. To this aim, healthcare (i.e., primary care) and societal (i.e., life insurance companies, etc.) systems should go beyond the sole use of the current BMI classification system to determine weight status in the general population, and consider alternative low-cost measures easily available in any primary care setting that can be used in combination with BMI, such as waist-to-height ratio (≥0.55) [53]. Regarding this point, future research is still needed in several directions, for instance, improving BMI performance by developing a modified BMI adjusted formula (i.e., age, sex, muscle and fat mass, etc.) or identifying new simple-to-use tools based on traditional techniques such as anthropometry and bioelectrical impedance analysis. Additionally, new techniques based on digital imaging or scanning could be employed [54] to precisely estimate body composition compartments, and thus be a more reliable way of classifying an individual’s weight status. Moreover, other works should extend the aim of our analysis to other European countries (i.e., Central, Eastern, Northern, Southern, and Western Europe), as well as on a global scale (Asia, Middle and Far East, and South and North America).

## 5. Conclusions

Disagreement exists between WHO BMI and DXA-derived BF% classification systems in determining weight status in the general population among all body weight ranges and age groups of both genders. One in three adults among the Italian general population is misclassified and placed in an incorrect weight status category, resulting in an overestimation of the prevalence of underweight, overweight, and obesity. Accordingly, public health guidelines in Italy need to be revised to consider combining direct body composition or their surrogate measures (i.e., skinfold measurement, body circumferences, etc.) with BMI while assessing weight status in the general population.

## Figures and Tables

**Table 1:** Anthropometric characteristics of the study participants.

	Total (*n* = 1351)	Males (*n* = 541)	Females (*n* = 810)	Significance
Age (year)	45.5 (21.4)	49.7 (21.7)	42.7 (20.7)	*p* < 0.001 ^γ^
Weight (kg)	69.6 (13.8)	77.7 (11.8)	64.3 (12.3)	*p* < 0.001 ^¥^
Height (cm)	166.9 (8.7)	173.6 (6.2)	162.4 (7.2)	*p* < 0.001 ^γ^
BMI (kg/m^2^)	25.0 (4.6)	25.8 (3.9)	24.5 (5.0)	*p* < 0.001 ^γ^
ALM (kg)	20.5 (4.9)	25.1 (3.5)	17.4 (2.9)	*p* < 0.001 ^γ^
Body fat (kg)	19.4 (8.4)	17.8 (7.4)	20.5 (8.8)	*p* < 0.001 ^γ^
Body fat (%)	27.7 (8.7)	22.5 (6.7)	31.2 (8.1)	*p* < 0.001 ^γ^

All values are mean (SD) for continuous variables and *n* (%) for categorical variables; ALM = appendicular lean mass. ^γ^  *p* values for Welch’s test for mean comparison. ^¥^  *p* values for Student’s *t*-test for mean comparison.

**Table 2:** Distribution of body weight status by the two classification systems (*n* = 1351).

	BMI Classification ^¥^	Body Fat (%) Classification ^£^
Underweight	19 (1.4)	84 (6.2)
Normal weight	787 (58.3)	773 (57.2)
Overweight	354 (26.2)	316 (23.4)
Obesity	191 (14.1)	178 (13.2)

^¥^ Based on WHO criteria for classification of BMI. ^£^ Body fat classification is based on sex and ethnicity specific cut-off points specified by Gallagher et al., 2000 [27].

**Table 3:** Agreement of the classification of body weight status between the two classification systems in the overall sample (*n* = 1351) *.

	BMI Classification						
BF% Classification	Underweight	Normal Weight	Overweight	Obesity	Total	Chi-Squared	Weighted Kappa
Under fat	6 (31.6%)	76 (9.7%)	2 (0.6%)	0 (0.0%)	84 (6.2%)	X^2^ = 884.17; *p* < 0.001	0.126, *p* < 0.0001
Normal fat	13 (68.4%)	615 (78.1%)	141 (39.8%)	4 (2.1%)	773 (57.2%)		
Over fat	0 (0.0%)	90 (11.4%)	165 (46.6%)	61 (31.9%)	316 (23.4%)		
Excess fat	0 (0.0%)	6 (0.8%)	46 (13.0%)	126 (66.0%)	178 (13.2%)		

* All values are *n* (%); BMI = body mass index; BF% = body fat percentage.

**Table 4:** Distribution of misclassification by sex, age group, and BMI status among Italian adults (*n* = 1351) *.

	Males (*n* = 541)		Females (*n* = 810)		Total (*n* = 1351)	
Age Group	Correctly Classified	Misclassified	Correctly Classified	Misclassified	Correctly Classified	Misclassified
Overall sample						
Total	364 (67.3%)	177 (32.7%)	548 (67.7%)	262 (32.3%)	912 (67.5%)	439 (32.5%)
18–39	157 (43.1%)	59 (33.3%)	301 (54.9%)	126 (48.1%)	458 (50.2%)	185 (42.1%)
40–59	34 (9.3%)	20 (11.3%)	107 (19.5%)	41 (15.6%)	141 (15.5%)	61 (13.9%)
60–79	161 (44.2%)	92 (52.0%)	129 (23.5%)	79 (30.2%)	290 (31.8%)	171 (39.0%)
80+	12 (3.3%)	6 (3.4%)	11 (2.0%)	16 (6.1%)	23 (2.5%)	22 (5.0%)
BMI < 18.5 kg/m^2^						
Total	0(0.0)	4 (100)	6 (40.0)	9 (60.0)	6 (31.6)	13 (68.4)
18–39	0(0.0)	1(100)	5 (35.7)	9 (64.3)	5 (33.3)	10 (66.7)
40–59	0(0.0)	0(0.0)	1 (100.0)	0(0.0)	1(100)	0(0.0)
60–79	0(0.0)	3(100)	0(0.0)	0(0.0)	0(0.0)	3(100)
80+	0(0.0)	0(0.0)	0(0.0)	0(0.0)	0(0.0)	0(0.0)
18.5 ≤ BMI < 25 kg/m^2^						
Total	219 (83.3)	44 (16.7)	396 (75.6)	128 (24.4)	615 (78.1)	172 (21.9)
18–39	136 (90.1)	15 (9.9)	262 (76.6)	80 (23.4)	398 (80.7)	95 (19.3)
40–59	17 (89.5)	2 (10.5)	53 (71.6)	21 (28.4)	70 (75.3)	23 (24.7)
60–79	64 (72.7)	24 (27.3)	73 (77.7)	21 (22.3)	137 (75.3)	45 (24.7)
80+	2 (40.0)	3 (60.0)	8 (57.1)	6 (42.9)	10 (52.6)	9 (47.4)
25 ≤ BMI < 30 kg/m^2^						
Total	94 (47.7)	103 (52.3)	71 (45.2)	86 (54.8)	165 (46.6)	189 (53.4)
18–39	15 (28.3)	38 (71.7)	16 (32.7)	33 (67.3)	31 (30.4)	71 (69.6)
40–59	8 (38.1)	13 (61.9)	25 (71.4)	10 (28.6)	33 (58.9)	23 (41.1)
60–79	64 (56.6)	49 (43.4)	28 (42.4)	38 (57.6)	92 (51.4)	87 (48.6)
80+	7 (70.0)	3 (30.0)	2 (28.6)	5 (71.4)	9 (52.9)	8 (47.1)
BMI ≥ 30 kg/m^2^						
Total	51 (66.2)	26 (33.8)	75 (65.8)	39 (34.2)	126 (66.0)	65 (34.0)
18–39	6 (54.5)	5 (45.5)	18 (81.8)	4 (18.2)	24 (72.7)	9 (27.3)
40–59	9 (64.3)	5 (35.7)	28 (73.7)	10 (26.3)	37 (71.2)	15 (28.8)
60–79	33 (67.3)	16 (32.7)	28 (58.3)	20 (41.7)	61 (62.9)	36 (37.1)
80+	3 (100.0)	0 (0.0)	1 (16.7)	5 (83.3)	4 (44.4)	5 (55.6)

* All values are *n* (%); BMI = body mass index.

## Data Availability

The original contributions presented in this study are included in the article. Further inquiries can be directed to the corresponding author.
